# Visual Impairment and Objectively Measured Physical Activity in Middle-Aged and Older Adults

**DOI:** 10.1093/geroni/igab046.1300

**Published:** 2021-12-17

**Authors:** Yurun Cai, Jennifer Schrack, Jian-Yu E, Amal Wanigatunga, Jacek Urbanek, Eleanor Simonsick, Bonnielin Swenor

**Affiliations:** 1 Johns Hopkins Bloomberg School of Public Health, Baltimore, Maryland, United States; 2 Johns hopkins Bloomberg School of Medicine, Baltimore, Maryland, United States; 3 Johns Hopkins School of Medicine, Baltimore, Maryland, United States; 4 National Instute on Aging/NIH, Baltimore, Maryland, United States

## Abstract

Vision loss is associated with restricted physical activity (PA), yet the relationship between multiple domains of vision measures and objectively measured PA, especially activity patterns, in mid-to-late life remains unclear. In 603 BLSA participants (mean age=73.5±11 years; 56% women; 69% white), best-corrected and presenting visual acuity (VA), contrast sensitivity, visual fields (VF), stereo acuity were assessed from 2015 to 2019. Free-living PA was assessed using a wrist-worn ActiGraph accelerometer for 7 days. Linear regression models showed that participants with vs. without best-corrected VA impairment had 29.3 fewer active minutes/day (p=0.03) and trended towards fewer activity counts (p=0.05), adjusting for sociodemographic and health characteristics. VF impairment was associated with 268,636 fewer activity counts (p=0.02), 46.2 fewer active minutes/day (p=0.02), and a 3% greater activity fragmentation (p=0.009). Older adults with visual impairment have restricted and more fragmented activity patterns. Longitudinal studies are warranted to examine causality between visual impairment and PA decline.

